# Physical Activity in Non-Frail and Frail Older Adults

**DOI:** 10.1371/journal.pone.0123168

**Published:** 2015-04-24

**Authors:** F. Marijke Jansen, Rick G. Prins, Astrid Etman, Hidde P. van der Ploeg, Sanne I. de Vries, Frank J. van Lenthe, Frank H. Pierik

**Affiliations:** 1 Department of Urban Environment and Safety, TNO, Utrecht, The Netherlands; 2 Department of Public Health, Erasmus MC, Rotterdam, The Netherlands; 3 Department of Public and Occupational Health, EMGO Institute for Health and Care Research, VU University Medical Center Amsterdam, Amsterdam, The Netherlands; 4 Department of Healthy Living, TNO, Leiden, The Netherlands; 5 Healthy Lifestyle in a Supporting Environment, The Hague University of Applied Sciences, The Hague, The Netherlands; 6 Human geography and Planning, Utrecht University, Utrecht, The Netherlands; Medical University Vienna, AUSTRIA

## Abstract

**Introduction:**

Physical activity (PA) is important for healthy ageing. Better insight into objectively measured PA levels in older adults is needed, since most previous studies employed self-report measures for PA assessment, which are associated with overestimation of PA.

**Aim:**

This study aimed to provide insight in objectively measured indoor and outdoor PA of older adults, and in PA differences by frailty levels.

**Methods:**

Data were collected among non-frail (N = 74) and frail (N = 10) subjects, aged 65 to 89 years. PA, measured for seven days with accelerometers and GPS-devices, was categorized into three levels of intensity (sedentary, light, and moderate-to-vigorous PA).

**Results:**

Older adults spent most time in sedentary and light PA. Subjects spent 84.7%, 15.1% and 0.2% per day in sedentary, light and moderate-to-vigorous PA respectively. On average, older adults spent 9.8 (SD 23.7) minutes per week in moderate-to-vigorous activity, and 747.0 (SD 389.6) minutes per week in light activity. None of the subjects met the WHO recommendations of 150 weekly minutes of moderate-to-vigorous PA. Age-, sex- and health status-adjusted results revealed no differences in PA between non-frail and frail older adults. Subjects spent significantly more sedentary time at home, than not at home. Non-frail subjects spent significantly more time not at home during moderate-to-vigorous activities, than at home.

**Conclusions:**

Objective assessment of PA in older adults revealed that most PA was of light intensity, and time spent in moderate-to-vigorous PA was very low. None of the older adults met the World Health Organization recommendations for PA. These levels of MVPA are much lower than generally reported based on self-reported PA. Future studies should employ objective methods, and age specific thresholds for healthy PA levels in older adults are needed. These results emphasize the need for effective strategies for healthy PA levels for the growing proportion of older adults.

## Introduction

According to the World Health Organization (WHO), by 2050 the percentage of people aged 65 years and older is expected to be doubled to the figure of 16%, and the number of people aged over 80 years will increase by 315% (double ageing) [1]. Ageing and age related diseases have great impact on society. The individual and societal impact of ageing can be reduced by stimulating PA, since PA promotes healthy ageing [2]. In particular for older adults, PA prevents falling, improves fitness and quality of life, contributes to prevention of osteoporosis, helps older adults to maintain their daily functioning, and decreases the risk of all-cause mortality [3, 4].

Ageing comes with the loss of muscle mass and strength [5]. This phenomenon, called sarcopenia, is highly prevalent among older adults and has been identified as an important risk factor for frailty [5]. Frailty is a state of vulnerability with an increased risk of adverse health outcomes, caused by deterioration of bodily functions [6]. It is of special interest as it puts a high demand on medical and social care [7]. Of older adults aged 65 to 74 years, 10.7% are frail [8]. Frail older adults are at increased risk of disabilities, fall incidents, hospitalization and death [6]. Frailty is associated with low levels of PA [9].

According to the WHO, health benefits can be achieved by older adults aged 65 years and older, when they accumulate at least 150 minutes of moderate intensity aerobic PA (MPA), or at least 75 minutes of vigorous intensity aerobic PA (VPA), or an equivalent combination of both intensities [4].

In addition, research has shown that also light PA (LPA) [10] and overall increased energy expenditure [11] positively affects health in older adults. However, data on various levels of PA and data on the prevalence of older adults meeting PA recommendations are limited [12]. Sedentary behaviour is of special interest, as it is negatively associated with health even when subjects do meet recommendations for MVPA [13].

Most studies investigating PA in older adults used self-report measures, which suffer from limitations such as recall bias, socially desirable responses, and the influence of factors as mood and cognition [12,14]. Many people tend to overestimate their level of PA. Watkinson et al. (2010), found that nearly half of objectively classified inactive individuals overestimated PA, and reported to be active [15]. Tucker, Welk and Beyler (2011) compared self-reported and objective measurements and found that compliance with PA guidelines was 62% according to self-report measures, while this was only 9.6% according to accelerometry [16]. Overestimating PA levels might be a barrier to behaviour change, because people are not aware that they are not physically active enough. Since levels of PA reported by studies using self-report measures might be biased, more studies investigating PA objectively are needed [12]. This study addresses this need by investigating light PA and MVPA, metabolic equivalent of task (MET) minutes, and active transportation among older adults. Further, we explore whether there are differences according to level of frailty.

Moreover, it is important to gain insight in locations where older adults are physically active. Previous studies have shown that older adults who are physically active outside, spend more time in MVPA than people who are only physically active inside [17]. Spending time outdoors might have additional benefits, since it positively affects cognitive functioning [18]. Both PA and being outdoors are associated with higher self-reported physical functioning, less fear of falling and less symptoms of depression [19]. But also indoor PA, such as cleaning and kitchen work, contributes to health and has shown to reduce all-cause mortality [20]. For older adults, especially the frail, active transportation might be an important source of PA since it is feasible for many individuals and it requires no specific skills. Despite their differential effects on health, indoor and outdoor PA are often not studied separately. Recently, this led Kerr et al. (2012) to suggest that researchers should compare indoor and outdoor PA, using GPS-devices and accelerometers instead of self-report instruments [17].

In sum, more objectively collected evidence of PA levels in older adults is needed to provide a baseline for international comparisons, to inform public health policies [12], and to enhance interventions targeted at improving lifestyle and health status of older adults. Therefore, the aim of this study was to describe indoor and outdoor PA in older adults, using GPS and accelerometers, and to assess differences in PA between non-frail and frail older adults.

## Methods

### Study design

This cross-sectional study was part of ELANE (ELderly And their NEighborhood), a study that was conducted in a middle-sized city (Spijkenisse) in the Netherlands, to investigate associations between environmental characteristics and PA, functional loss, independent living and quality of life. ELANE employed the same research structure and methods as the Prevention and Reactivation Care Program study (PreCAP), that was conducted in the same study area [21]. A more detailed description of the ELANE study design is provided elsewhere [22]. The research has been conducted with the approval of the Medical Ethics Committee of the Erasmus Medical Center Rotterdam (METC Erasmus MC).

### Participants and setting

**Fig 1:**
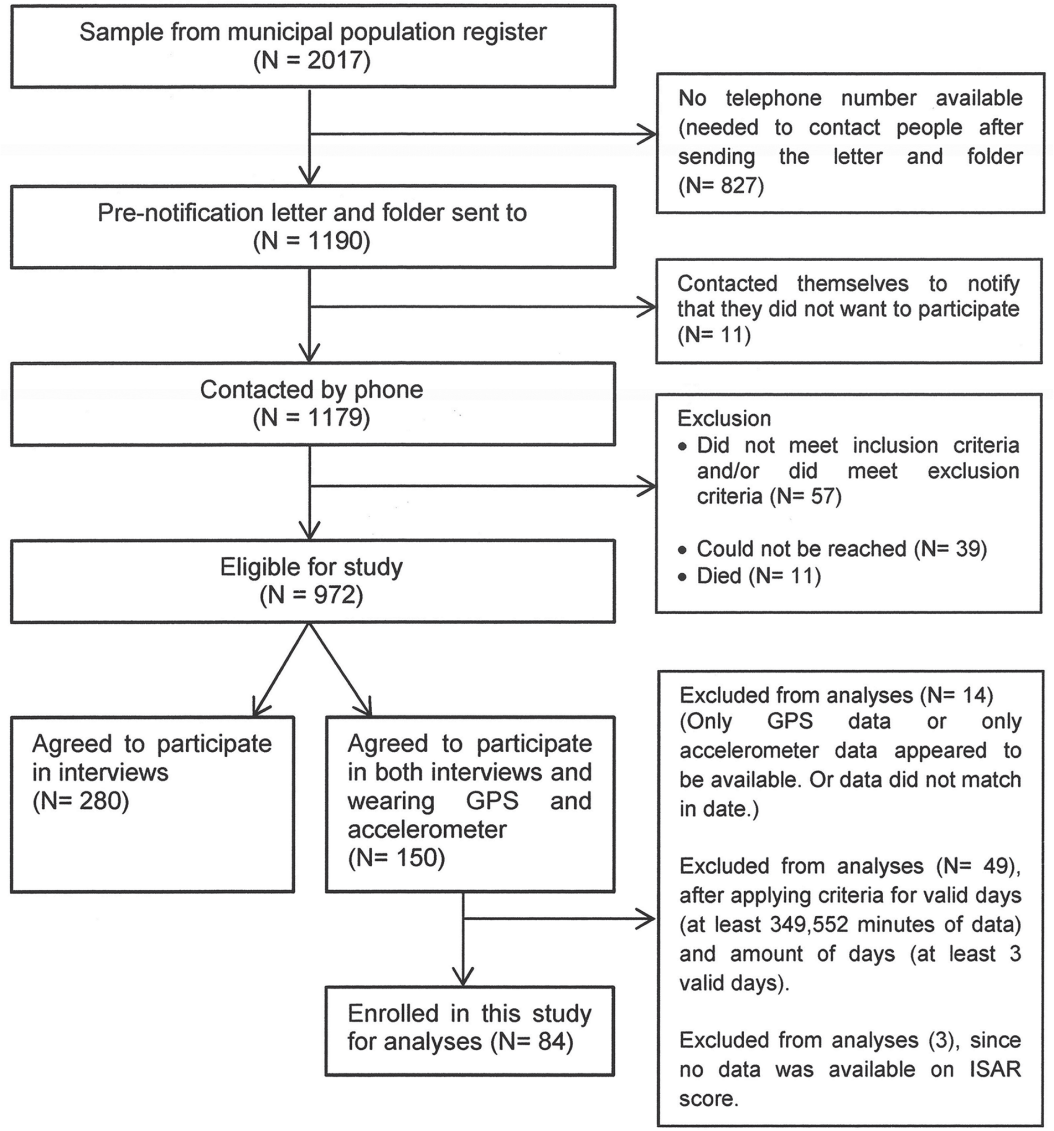
Flow chart of participant recruitment.

The flowchart in Fig 1 illustrates the recruitment- and selection procedure. Community-dwelling older adults, recruited from the municipal population register of Spijkenisse (N = 2017), were initially informed by using a pre-notification letter and information brochure. Subsequently they were contacted by phone and during the phone conversation it was determined whether persons met the inclusion criteria: aged 65 years and older, and master the Dutch language sufficiently. Exclusion criteria were: living in a residential care or nursing home, recent admission to a hospital, bound to wheelchair or mobile scooter, or being bedridden. Older adults who were eligible for participation (N = 972) were asked to participate in an interview, and to wear an accelerometer and a GPS-device. Of the 972 older adults, 430 (44.2%) were willing to participate in ELANE. All 430 individuals participated in the interview, and among them, 150 older adults were willing to wear the accelerometer and GPS-device. The present study used data of this subsample of 150 older adults (34.9%). All participants have given informed consent. During a home visit, trained staff-members conducted an interview (based on a questionnaire) and distributed the accelerometer and GPS device. Data was collected between September 2011 and June 2012. After applying criteria for valid accelerometer and GPS data, 84 participants were included for analyses. There were no significant differences in age, sex, frailty, marital status, and health status between participants who were excluded from analyses and the final study sample.

### Assessment of PA

PA was measured objectively by using ActiGraph GT3X+ (ActiGraph, Pensacola, Florida) accelerometers. Participants were instructed to wear the accelerometer at the right hand side of the waist, during waking hours (except during water-based activities) for seven consecutive days. A manual was handed out, to read back instructions. The manufacturers software (ActiLife v5.8.3 Firmware v2.2.0, ActiGraph) was used to process accelerometer data. In this study population, sequences of 60 minutes or more of consecutive zero counts were excluded from each recording, as these were considered to be non-wear time [14, 16].

In order to have sufficient data for a reliable estimate of PA, recommendations have been made to include only participants with sufficient data on a daily basis, and to have at least three valid days of data [23]. In accordance with these recommendations, a valid day of accelerometer data was defined as having non-missing counts for at least 80% of a measurement day [24]. A measurement day was defined as the time in which at least 70% of participants wore the device [24], which was in this study 437 minutes. Hence, each day in which a participant had data for 80% of the measurement day, (i.e. 437*0.8 = 394.5 minutes) was considered a valid measurement day. Every participant with at least three of such valid measurement days was included in the analyses.

Accelerometer data were collected in ten second epochs and aggregated as counts per minute (cpm). After cleaning the data, counts per minute were classified in different intensities of PA. For older adults there is no consensus for cut-off values in cpm [14]. Cut-off values for this study were therefore based on studies with similar study populations [25–27]. Sedentary, light, and moderate-to-vigorous PA levels were defined as 0–50 cpm, 51–759 cpm, and 760–1951 cpm, and ≥ 1952 cpm respectively. MVPA was calculated as the sum of moderate and vigorous PA. Although activities of 0–50 cpm are classified as ‘sedentary’ throughout this paper, this category may also include other situations when the subject may not be seated, but her or his PA level is equivalent to someone in a seated position.

WHO PA recommendations [4] are met when one is physically active for ≥ 150 minutes with moderate-intensity per week, ≥ 75 minutes with vigorous-intensity per week, or an equivalent combination of both intensities. To assess this, one minute of vigorous PA (VPA) can be considered to be equal to two minutes of moderate PA (MPA) [16]. In the current paper, daily mean time in MVPA (MPA + 2xVPA) was multiplied by seven to derive the time spent in MVPA per week. Participants met the recommendations if they accumulated ≥ 150 minutes MVPA per week.

In order to calculate energy expenditure from accelerometer data, METs were derived from cpm using the following regression equation: 1.439008 + 0.000795*counts/min [28]. These MET minutes were added up and divided by the number of valid measurement days. This gave the mean daily MET minutes. Weekly MET minutes were computed by multiplying mean daily MET minutes by seven, and include energy expenditure in sedentary behaviour, light intensity activities and moderate-to-vigorous intensity activities.

### Assessment of PA location

GPS receivers BT-Q1000X and BT-Q1000XT (QStarz International Co) were used to map geographical locations of participants. Participants were instructed to wear the GPS receiver on the same belt the accelerometer was placed on, at the right hand side of the waist, during waking hours (except during showering and swimming), for seven consecutive days. Locations were measured every ten seconds. GPS receiver data were mapped using the Qstarz QTravel software (v1.45 for Win XP/Vista/7) and it was date- and time-linked to the accelerometer data to create combinations of activity and location. In this study, PA at home was compared to PA at other locations (both indoors and outdoors), referred to as not at home. Identification of ‘at home’ and ‘not at home’ was performed using cluster detection on GPS data [29]. In cluster detection, first all GPS data were projected on a grid with cell sizes of 25m x 25m. The cell that contained most GPS locations (>100) was determined. A list was made of all locations in this cell and in eight surrounding cells. From this list the center of gravity (COG) was calculated. Furthermore the root mean square (d_RMS_) of the distances of all GPS data points to the COG was calculated. Data points that were more than three d_RMS_ away from the COG are eliminated from the data list and marked as outliers. Then the COG is calculated again and if it lies more than five meters from the first COG, the outlier removal procedure was repeated. Data points that were less than two d_RMS_ away from the COG were marked as part of the cluster. All data points of the cluster corresponding to the home address were classified as ‘at home’.

### Assessment of active transportation distances

Daily transportation distances per active mode of transport were derived from GPS measurements. The mode of transport (i.e. walking or cycling) was defined based on the speed that was registered by the GPS device. For every outdoor data point, the maximum speed was determined, calculated as the maximum during the two-minute time interval that starts one minute before and ends one minute after the time stamp of the data point [30]. In this study, a data point was classified as ‘walking’ if the maximum speed was ≤ 9.0 km/h, and the data point was classified as ‘cycling’ if the maximum speed was > 9.0 km/h and ≤ 30 km/h. If the maximum speed was ≤ 1 km/h it was classified as ‘stationary’. These cut-off values for walking and cycling were defined by Prins et al. (2014) for an elderly study population [31]. Episodes (parts of GPS-tracks outdoors) of cycling or walking were defined as sequences of data points with a constant mode of transport. Episodes with a duration shorter than two minutes were considered to be too short to be valid, and the transportation mode of these episodes was changed to the transportation mode of the preceding or following episode (depending on which had the longest duration). All episodes of cycling of one day were summed to calculate daily cycling distance, and the episodes of walking of one day were summed to calculate daily walking distance.

### Assessment of personal characteristics

Information on age, sex, frailty and health status was collected during the interviews, using a questionnaire. Health status was measured using a question of the SF-12 questionnaire: ‘In general, would you say your health is’. Answer options were 1) Excellent, 2) Very good, 3) Good, 4) Fair, 5) Poor. Frailty status was determined using the ‘Identification Seniors At Risk—Hospitalized Patients’ questionnaire (ISAR-HP) to determine frailty, which has sufficient validity [32, 33]. The ISAR-HP assesses the presence or absence of predictors of adverse health outcomes and consists of four items: 1) help needed in instrumental activities of daily living (e.g. housekeeping, shopping), 2) use of walking device (e.g. cane, walker), 3) assistance needed for travelling, and 4) continued education after age 14 [33]. Items could only be answered with yes or no, and were scored as follows: item 1 and 3: yes = 1, no = 0; item 2: yes = 2, no = 0; item 4: yes = 0, no = 1. Based on results of this measurement, participants were classified as non-frail (score ≤ 1), or frail (score ≥ 2).

### Statistical analyses

Descriptive statistics were used to present baseline characteristics and to describe time spent at home and not at home in different PA levels per day. Paired samples *t*-tests were used to analyze differences in mean time spent at home and not at home. Most of the dependent variables were not normally distributed and neither log transformations nor taking the square root of variables, led to normal distributions. Therefore, bootstrapped linear regression was used (number of samples set to 5000) to assess differences in PA behaviour between non-frail and frail older adults. PA measures were regressed on the frailty indicator and adjusted for age [23, 27], sex [23, 27, 34], and health status. P-values <. 05 were considered statistically significant. SPSS 20.0 for Windows was used for statistical analyses.

## Results

Table 1 summarizes personal characteristics and levels of PA for the study population (N = 84), and for non-frail and frail older adults. Of the participants, 46.4% were female, and 11.9% of the older adults were classified as frail. The age of participants ranged from 65.3 to 89.6 years, with a mean age of 73.6 (SD 6.3) years. On average, men were aged 74.5 (SD 6.7) years, and women 72.5 (SD 5.7) years. Non-frail older adults had a mean age of 73.4 (SD 6.1) years, whereas frail older adults had a mean age of 74.4 (SD 7.6) years. The percentage of subjects that valued their health status to be good, very good or excellent was 86.5% for non-frail, and 30% for frail older adults. On average, accelerometers and GPS-devices were worn for 703.2 (SD 13.0) minutes per day. In total 416 days of 84 participants were included for analyses.

**Table 1:** General characteristics of participants.

Characteristics	Total study population (N = 84); N (%)	Non-frail (N = 74), ISAR score ≤ 1 ; N (%)	Frail (N = 10), ISAR score ≥ 2; N (%)
Sex			
Male	45 (53.6)	42 (56.8)	3 (30.0)
Female	39 (46.4)	32 (43.2)	7 (70.0)
Age (in years)			
Mean (SD)	73.6 (SD 6.3)	73.4 (SD 6.1)	74.4 (SD 7.6)
Minimum	65.3	65.3	65.7
Maximum	89.6	89.6	84.5
Age in categories			
65–74	54 (64.3)	49 (66.2)	5 (50.0)
75–84	26 (31.0)	21 (28.4)	5 (50.0)
≥ 85	4 (4.8)	4 (5.4)	0 (0.0)
Age (according to sex)			
Men	74.5 (SD 6.7)	74.3 (SD 6.4)	78.2 (SD 10.3)
Women	72.5 (SD 5.7)	72.3 (SD 5.6)	73.2 (SD 6.4)
Health status			
1 Excellent	7 (8.3)	6 (8.1)	1 (10.0)
2 Very good	16 (19.0)	15 (20.3)	1 (10.0)
3 Good	44 (52.4)	43 (58.1)	1 (10.0)
4 Fair	16 (19.0)	9 (12.2)	7 (70.0)
5 Poor	1 (1.2)	1 (1.4)	0 (0.0)
**Weekly PA**			
LPA per week (minutes)			
Mean (SD)	747.0 (SD 389.6)	751.1 (SD 393.9)	716.9 (SD 374.5)
Minimum	0.0	0.0	253.0
Maximum	1906.0	1906.0	1490.0
Median (Q1—Q3)	755.8 (455.3–946.5)	800.5 (464.7–943.1)	703.8 (377.7–954.6)
MVPA per week (minutes)			
Mean (SD)	9.8 (SD 23.7)	10.8 (SD 25.1)	2.8 (SD 3.1)
Minimum	0.0	0.0	0.0
Maximum	139.0	139.0	9.2
Median (Q_1_-Q_3_)	2.2 (0.6–7.2)	2.3 (0.7–9.2)	1.4 (0.5–5.3)
Met WHO recommendations			
Yes	0 (0.0)	0 (0.0)	0 (0.0)
No	84 (100.0)	74 (100.0)	10 (100.0)
MET minutes per week			
Mean (SD)	5511.8 (SD 1869.3)	5591.6 (SD 1869.6)	4921.7 (SD 1595.6)
Minimum	2197.5	2197.5	2805.2
Maximum	9219.0	9219.0	7962.9
Median (Q_1_-Q_3_)	5887.6 (3570.6–6809.0)	6022.5 (3482.7–6862.5)	4671.6 (3709.6–5996.1)
**Valid data**			
Average daily wear time (minutes)			
Mean (SD)	703.2 (SD 118.8)	704.7 (SD 120.1)	691.7 (SD 114.4)
Total days included in analyses			
(i.e. valid days)	416	365	51

Note: SD = standard deviation, Q_1_ = 25^th^ percentile, Q_3_ = 75^th^ percentile, MET = Metabolic Equivalent of Task.

### Weekly PA

On average, participants spent 9.8 (SD 23.7) minutes in MVPA per week [Table 1]. Non-frail older adults accumulated on average 10.8 (SD 25.1) minutes of MVPA, whereas frail older adults accumulated on average 2.8 (SD 3.1) minutes in MVPA. None of the participants met the WHO recommendations for aerobic PA. The older adults accumulated on average 747.0 (SD 389.6) minutes in LPA per week [Table 1]. Non-frail and frail older adults accumulated respectively 751.1 (SD 393.9) minutes and 716.9 (SD 374.5) minutes of LPA per week. No statistically significant differences were found in weekly LPA and weekly MVPA between non-frail and frail older adults. Also, no statistically significant differences in weekly MET minutes were found between non-frail and frail older adults, who accumulated on average 5591.6 (SD 1898.6) and 4921.7 (SD 1595.6) MET minutes per week, respectively [Table 1].

### Differences in daily time spent in activity intensities according to frailty categories

Time spent in different levels of PA is shown in Table 2 (mean and SD) and in Table 3 (median and interquartiles). Non-frail and frail older adults spent respectively 84.7% and 84.9% of the day sedentary. This equates to approximately ten hours per day. Less time is spent in light activities: 15.1% (non-frail) and 15.0% (frail) per day, which equates to almost two hours. Older adults of both frailty categories spent less than two minutes per day in MVPA. After adjustment for confounders (age, sex and health status), no significant difference was found between frailty categories.

**Table 2:** Time spent in different PA levels by frailty status (mean and SD).

PA	Total study population (N = 84)		Non-frail (N = 74) ; ISAR HP score ≤ 1		Frail (N = 10); ISAR HP score ≥ 2	
	Minutes (SD)	Percentage (SD)	Minutes (SD)	Percentage (SD)	Minutes (SD)	Percentage (SD)
Sedentary (0–50 cpm)	595.10 (113.57)	84.71 (7.69)	595.93 (112.98)	84.69 (7.67)	588.93 (123.94)	84.90 (8.25)
At home	374.38 (154.90)	52.00 (19.14)	370.32 (159.61)	51.07 (19.53)	404.42 (116.17)	58.87 (15.08)
Not at home	220.72^a^ (115.65)	32.72^a^ (20.23)	225.62^a^ (117.62)	33.62^a^ (20.99)	184.51^a^ (97.31)	26.03^a^ (12.14)
Light PA (51–759 cpm)	106.71 (55.65)	15.09 (7.53)	107.29 (56.27)	15.10 (7.49)	102.41 (53.50)	15.03 (8.20)
At home	53.74 (36.15)	7.49 (4.71)	54.19 (37.15)	7.49 (4.79)	50.41 (28.97)	7.43 (4.26)
Not at home	52.98 (33.32)	7.60 (4.88)	53.11 (32.92)	7.60 (4.77)	52.00 (38.06)	7.61 (5.92)
MVPA (≥ 760 cpm)	1.35 (3.30)	0.20 (0.50)	1.49 (3.50)	0.21 (0.53)	0.39 (0.43)	0.06 (0.07)
At home	0.19 (0.26)	0.03 (0.04)	0.19 (0.27)	0.03 (0.04)	0.15 (0.15)	0.03 (0.03)
Not at home	1.17^a^ (3.28)	0.17^a^ (0.49)	1.30^a^ (3.48)	0.19^a^ (0.52)	0.24 (0.32)	0.04 (0.05)

Note: Percentages represent average amount of time spent per day, based on daily wear time (missing values were excluded from analyses).

^a^Time spent not at home differs significantly from time spent at home in raw paired samples t-test (p<0.05).

**Table 3:** Time spent in different PA levels by frailty status (median and interquartiles).

PA	Total study population (N = 84)		Non-frail (N = 74) ; ISAR HP score ≤ 1		Frail (N = 10); ISAR HP score ≥ 2	
	Minutes (Q1-Q3)	Percentage (Q1-Q3)	Minutes (Q1-Q3)	Percentage (Q1-Q3)	Minutes (Q1-Q3)	Percentage (Q1-Q3)
Sedentary	596.80 (511.97–672.60)	85.25 (80.47–90.07)	596.80 (510.69–671.39)	84.83 (80.29–89.81)	598.11 (509.64–686.54)	86.73 (80.95–92.02)
At home	381.05 (281.37–503.46)	56.48 (40.76–64.18)	380.58 (273.52–507.78)	55.36 (40.60–63.72)	414.82 (317.69–497.79)	60.80 (50.73–70.17)
Not at home	201.87 (142.54–278.64)	29.95 (18.95–38.97)	201.87 (143.61–286.28)	30.27 (19.58–40.55)	185.74 (94.31–250.52)	26.26 (14.22–36.53)
Light PA	107.97 (63.61–135.21)	14.42 (9.93–19.24)	114.35 (66.38–134.73)	14.71 (10.18–19.28)	100.54 (53.95–136.37)	13.25 (7.96–19.04)
At home	48.95 (30.48–74.02)	6.78 (3.97–10.72)	49.36 (29.98–75.03)	6.78 (3.86–10.96)	45.45 (26.70–70.30)	6.62 (4.38–10.84)
Not at home	48.03 (28.61–73.47)	6.96 (4.15–9.74)	48.70 (29.23–74.17)	7.12 (4.17–9.80)	43.83 (26.12–60.26)	6.30 (3.84–8.10)
MVPA	0.31 (0.07–1.03)	0.04 (0.01–0.16)	0.32 (0.07–1.14)	0.05 (0.01–0.17)	0.18 (0.07–0.76)	0.02 (0.01–0.13)
At home	0.11 (0.02–0.28)	0.01 (0.00–0.04)	0.11 (0.02–0.27)	0.02 (0.00–0.04)	0.09 (0.02–0.31)	0.01 (0.00–0.05)
Not at home	0.12 (0.02–0.49)	0.02 (0.00–0.07)	0.12 (0.02–0.53)	0.02 (0.00–0.07)	0.14 (0.02–0.37)	0.02 (0.00–0.07)

Note: Percentages represent average amount of time spent per day, based on daily wear time (missing values were excluded from analyses). Q_1_ = 25^th^ percentile, Q_3_ = 75^th^ percentile.

Time spent at home and not at home is shown in Table 2 (mean and SD) and 3 (median and interquartiles). Both non-frail and frail older adults engage significantly more time in sedentary behaviour at home than not at home. Non-frail older adults spent 51.1% (SD 19.5) of their time at home sedentary, and 33.6% (SD 21.0) when not at home. Frail older adults spent 58.9% (SD 15.1) of their time at home sedentary, and 26.0% (SD 12.1) not at home. Only non-frail older adults spent significantly more time per day in moderate-to-vigorous intensity not at home (1.3 minutes, SD 3.5), as compared to at home (0.2 minutes, SD 0.3).

### Active transportation distances

Older adults walked on average 2486.9 (SD 1707.6) meters per day, and they cycled on average 3915.1 (SD 5215.8) meters per day [Table 4]. Walking and cycling distances did not differ significantly between the frailty groups [Table 5].

**Table 4:** Distance of active transportation per day.

	Total study population (N = 84)	Non-frail (N = 74) ; ISAR HP score ≤ 1	Frail (N = 10); ISAR HP score ≥ 2
Distance of active transportation—Walked			
Mean (SD)	2486.9 (SD 1707.6)	2534.2 (SD 1713.4)	2136.4 (SD 1709.8)
Median (Q1-Q3)	2107.1 (1316.9–3315.7)	2263.4 (1323.6–3346.1)	1805.3 (755.0–2862.1)
Distance of active transportation—Cycled			
Mean (SD)	3915.1 (SD 5215.8)	3871.0 (SD 5023.2)	4241.9 (SD 6788.5)
Median (Q1-Q3)	2341.4 (1022.7–4579.5)	2461.1 (1070.1–4617.6)	1503.5 (289.7–5216.1)

Note: Q_1_ = 25^th^ percentile, Q_3_ = 75^th^ percentile.

**Table 5:** Adjusted regression coefficients for time spent at different PA levels and active transportation distances, by frailty status.

PA	Frail vs. non-frail older adults ; (min)				Frail vs. non-frail older adults; (%)			
	B	95% CI for B		P-value	B	95% CI for B		P-value
		Lower	Upper			Lower	Upper	
Sedentary	-5.499	-88.958	63.011	0.873	0.248	-5.249	4.897	0.919
At home	15.876	-70.383	95.770	0.675	4.705	-6.107	14.971	0.371
Not at home	-21.374	-91.540	40.868	0.507	-4.458	-13.909	4.715	0.328
Light	-4.455	-35.132	32.826	0.782	-0.139	-4.428	5.028	0.956
At home	-4.877	-22.739	16.351	0.599	-0.307	-2.787	2.723	0.824
Not at home	0.422	-20.700	27.990	0.975	0.167	-2.929	4.450	0.927
MVPA	-0.716	-1.867	0.418	0.194	-0.110	-0.309	0.045	0.219
At home	-0.007	-0.101	0.087	0.870	-0.003	-0.014	0.020	0.748
Not at home	-0.710	-1.839	0.385	0.194	-0.112	-0.310	0.052	0.216
Weekly LPA	-31.183	-249.189	219.499	0.790	-	-	-	-
Weekly MVPA	-5.214	-13.778	3.116	0.187	-	-	-	-
Weekly MET minutes	-645.342	-1720.834	475.839	0.221	-	-	-	-
Active travel distance—walked	-542.578	-1566.148	749.596	0.336	-	-	-	-
Active travel distance—cycled	791.472	-3112.177	5704.442	0.750	-	-	-	-

Note: Adjusted for age, sex and health status. Non-frail was the reference category.

## Discussion

### Main findings and interpretation

The present study found that older adults spent only 9.8 (SD 23.7) minutes per week on MVPA. Since there is no consensus in accelerometer cut-off values to determine levels of PA for older adults, results of this study should be compared to other studies with caution. For example, Gorman et al. (2014) found in their review eight different cut-off values that were used for the classification of MVPA in older adults [14]. Due to this wide range of cut-off values, they found that reported medians of MVPA ranged from 4 to 80 minutes per day [14].

Besides, the present study found that none of the older adults met WHO recommendations for aerobic PA. These findings are inconsistent with national figures of PA of Dutch older adults: 75% of men and 67% of women aged 65–75 years, and 45% of older adults aged over 75 years, were physically active for at least 30 minutes of MVPA per day, on five days of a week [35]. However, these prevalence data were based on self-reported questionnaires, whereas the current study used accelerometers. Previous accelerometer-based studies examining PA behaviour of older adults, also reported much lower compliance with WHO recommendations than when assessed by self-report [12]. It is known that people tend to over-estimate their PA levels. Accurate assessment of PA levels is important, as misclassification can bias research results and conclusions. Moreover, an accurate classification of meeting the criteria of PA is essential to identify those who may benefit from an intervention. This indicates that there is a clear need for large scale, population-based studies using objective assessment of PA in older adults that, like the current study, differentiate PA in distinct levels. One could argue whether currently available cut-off values for MVPA are appropriate for elderly populations. For older adults, it might be more appropriate to develop age specific cut-off values [14], based on studies that relate levels of objectively measured PA (e.g. based on accelerometer counts or energy expenditure) to health status.

Although older adults spent only little time in moderate-to-vigorous activities, a considerable amount of time is spent in light activities. Light intensity activities often include activities of daily life (e.g. household, walking). No specific skills are required and therefore these activities can be easily integrated in older adults’ daily life’s. Current guidelines for PA have not yet integrated recommendations on LPA.

Furthermore, this study showed that older adults spent most time per day sedentary (84.7%), and this confirms findings of previous studies which reported that adults spent on average 62% to 86% per day sedentary [14]. Since sedentary behaviour has negative effects on health [13], it is of great importance to reduce time spent sedentary for example by stimulating LPA, since activities with light intensity have been identified as health resources [36].

Moreover, the results indicate that sedentary behaviour is more performed at home than not at home, whereas MVPA is more performed not at home. Although in this study little time is spent in MVPA, our results seem to indicate that spending time not at home might contribute to an increase of time spent in moderate-to-vigorous activity. This is consistent with literature, which shows that spending time outdoors might contribute to the frequency, duration and intensity of PA [17]. Moreover, adults are more likely to be physically active when they spend more time outdoors [17]. Therefore, activity friendly neighbourhoods and interventions offering outdoor activities, may increase time spent in light and moderate-to-vigorous PA in older adults.

In this study, additional analyses were conducted to calculate weekly energy expenditure in MET minutes. This study provides insight in objectively measured weekly energy expenditure of non-frail and frail older adults, and we did not find differences between frail and non-frail older adults with regard to MET minutes. However, more research is needed to draw strong conclusions.

This study showed that non-frail older adults had a higher daily walking distance than frail older adults, whereas the frail adults had a higher daily cycling distance. However, these differences were not statistically significant. In a previous study [31] it was shown that frail older adults travelled significantly shorter distances to do their groceries than non-frail elderly, but that the frequency of trips was higher. It seems that over the day, frail and non-frail older adults accumulate the same distances by active transportation, but that the distances per trip are lower for frail elderly (and hence proximity of facilities may be more important for them).

Findings of this study on cycling distances were comparable to previous findings in this age group in the Netherlands [37]. The bicycle is a very popular mode of transport in the Netherlands, especially for travel distances up to 7.5 kilometres [38]. The percentage of people who use a bicycle is higher in the Netherlands than in other European countries, and the average cycling distance is highest in the Netherlands as compared to other European countries. This popularity of bicycle use can partly be explained by the good cycling infrastructure, the compact Dutch cities and the flat landscape [38].

In this study, the prevalence of frail older adults was 11.9%, which is in accordance with previous findings. According to the review of Collard et al. (2012), the average prevalence of frailty among adults aged 65 years and older, was 10.7% (with a range from 4.0% to 59.1%) [8]. In this study, no significant differences in PA behaviour were found between frailty groups (after adjustment for confounding). This may be partly due to the small sample size, and differences might be significant when frailty groups were larger, or defined differently.

### Strengths and limitations

A major strength of this study is the use of accelerometers and GPS devices, which supplies high quality data. Accelerometers and GPS devices provide objective information on PA and are used to overcome limitations of self-report (e.g. recall bias, socially desirable answers) [12,14]. However, inaccuracy might occur in GPS devices due to surrounding obstacles, and accelerometers are less accurate in measuring upper body movements. This study has some limitations, such as the identification of frailty, the relatively small sample size, data loss, and possible selection bias. To identify non-frail, and frail older adults, the ISAR-HP instrument was used. As the ISAR-HP focuses on patients, this might not be the best instrument to determine frailty in community-dwelling older adults. There are other frailty measures, e.g. the Tilburg Frailty Indicator or Fried’s Phenotype, using a broader set of indicators to assess frailty. However, it is unclear whether the use of another measure would have changed the findings of the current study.

Taraldsen et al. (2012) reported that most studies that used accelerometers, had small sample sizes (≤ 50 participants) [39]. Although our study had a sample size of 84 participants, there are some studies with larger sample sizes, providing more powerful results [12, 14]. In this study, the separate frailty groups are still relatively small. To add more power to the analyses, it is desirable to have larger frailty groups and therefore a larger study population is required. The number of days the accelerometers and GPS devices were worn by participants was often insufficient, and therefore data of 44% of the older adults who participated in this study had to be excluded from analyses. In total 416 days (of 84 participants) could be included for analyses. The average daily wear time of these days was 703.2 (SD 118.8) minutes, which is nearly twelve hours per day.

Participants were randomly approached for participation, but only 15.4% of those eligible for participation, participated in this study and wore an accelerometer and GPS-device. Therefore, this study population may not be representative for the Dutch population, and we cannot rule out selection bias. However, in terms of age and sex distribution, our final study sample is representative for the population of Spijkenisse and for the Dutch population. Mean age of our final study sample was similar to the mean age of the population of Spijkenisse and the Dutch population, aged 65 years and older. Also, participants of the final study population were homogeneously distributed over Spijkenisse. People who volunteer for participation in an exercise study are often healthier and fitter than non-volunteers [40]. This might have led to an overestimation of PA.

Ethnicity and weather conditions (e.g. rainfall, temperature) are sometimes taken into account as confounders in studies assessing PA [16, 23, 34]. However, as no information on ethnicity was obtained in this study, it is not taken into account in the analyses. Additional analyses showed that the amount of rain, temperature, and windspeed did not differ significantly between non-frail and frail older adults in this study, and were therefore not taken into account as confounders.

## Conclusion

We conclude that PA levels of older adults aged 65 years and older were very low, and that most PA was of light intensity. In an ageing population health gains can be achieved by reducing sedentary behaviour and stimulating PA. More large scale population-based studies that use objective methods are needed to inform public health policy adequately. This study emphasizes the need for interventions aimed at stimulating healthy PA levels in older adults.

## Supporting Information

S1 Dataset Dataset underlying the findings in the manuscript in a spreadsheet. (XLS)
